# Yes-associated protein promotes the abnormal proliferation of psoriatic keratinocytes via an amphiregulin dependent pathway

**DOI:** 10.1038/s41598-018-32522-y

**Published:** 2018-10-15

**Authors:** Jinjing Jia, Changji Li, Jiao Yang, Xin Wang, Ruilian Li, Suju Luo, Zhengxiao Li, Jiankang Liu, Zhi Liu, Yan Zheng

**Affiliations:** 1 Department of Dermatology, the Second Affiliated Hospital of Guangzhou University of Chinese Medicine, Guangzhou, China; 2 Department of Dermatology, the Second Affiliated Hospital of Xi’an Jiaotong University, School of Medicine, Xi’an, China; 3 Department of Dermatology, Tianjin Medical University General Hospital, Tianjin, China; 4 Key Laboratory of Biomedical Information Engineering of the Ministry of Education, School of Life Science and Technology, Xi’an Jiaotong University, Xi’an, China; 5 Frontier Institute of Science and Technology, Xi’an Jiaotong University, Xi’an, China; 6 Department of Dermatology, University of North Carolina at Chapel Hill, Chapel Hill, NC 26599 USA

## Abstract

Psoriasis is a chronic inflammatory skin disease with high morbidity, poor treatment methods and high rates of relapse. Keratinocyte hyperproliferation and shortened cell cycles are important pathophysiological features of psoriasis. As a known oncogene, Yes-associated protein (YAP) plays a role in promoting cell proliferation and inhibiting cell apoptosis; however, whether YAP is involved in the pathogenesis of psoriasis remains to be determined. Amphiregulin (AREG), a transcriptional target of YAP, was found to be upregulated in psoriasis, and overexpression of AREG promoted keratinocyte proliferation. In the present study, immunohistochemistry showed that YAP expression was elevated in the skin of psoriasis patients and in the Imiquimod (IMQ) mouse model of psoriasis. Knockdown of YAP in HaCaT cells inhibited cell proliferation, caused cell cycle arrest in G0/G1 phase and promoted apoptosis. These changes in YAP-knockdown HaCaT cells were related to changes in AREG expression. We concluded that YAP may play an important role in the regulation of abnormal keratinocyte proliferation via an AREG-dependent pathway and that YAP could be a new target in the treatment of psoriasis.

## Introduction

Psoriasis is a chronic inflammatory skin disease characterized by hyperproliferation and abnormal differentiation of epidermal keratinocytes, infiltration of inflammatory cells and hyperplasia of dilated superficial dermal vessels. Psoriasis has high incidence and relapse rates, but the treatment options are poor. The lesions are often located in exposed areas, causing significant psychological and social distress to the patients and affecting their health and quality of life. Currently, the aetiology and pathogenesis of psoriasis is unclear. It is believed that harmful external stimuli such as trauma, infection, drugs and mental stress in combination with changes in the genetic background and immune system changes cause infiltration of inflammatory cells and increased levels of inflammatory mediators, resulting in abnormal epidermal keratinocyte proliferation, and differentiation, and clinical psoriasis^1,2^. Because keratinocyte hyperproliferation and shortened cell cycles are important pathological features, and because psoriatic keratinocytes show resistance to apoptosis, it is critical to identify the causes of the apoptosis imbalance and cell cycle acceleration to better understand the pathogenesis of psoriasis.

Yes-associated protein (YAP), a key component in the Hippo pathway, was originally identified in studies in *drosophila*^3^. YAP plays an important role in cell proliferation and apoptosis, tissue growth and development, epithelial-mesenchymal transition (EMT), intercellular contact inhibition and stem cell self-renewal^4,5^. Recent studies noted that YAP may serve as an oncogene in a wide variety of human cancers, including hepatocellular carcinoma^6^, ovarian cancer^7^, lung cancer^8^, pancreatic cancer^9^, oral squamous cell carcinoma (SCC)^10^ and melanoma^11^. Our previous studies showed that YAP was highly expressed in cutaneous SCC (cSCC), and promoted cSCC progression by regulating cell proliferation, cell cycle, apoptosis, migration and invasion^12^. Although psoriasis shares some common features with cSCC, it remains unclear whether YAP plays a role in psoriasis. Amphiregulin (AREG), a ligand for epidermal growth factor receptor (EGFR), appears to be a transcriptional target of YAP. AREG expression is upregulated in psoriasis, and overexpression of AREG promotes keratinocyte proliferation^13,14^. In this study, we tested the hypothesis that YAP promotes the abnormal proliferation of keratinocytes through the upregulation of AREG. We examined YAP expression in the skin of psoriasis patients and in the Imiquimod (IMQ)-induced mouse model and assessed its influence on the proliferation, cell cycle and apoptosis of keratinocytes *in vitro*.

## Results

### YAP protein expression is upregulated in the psoriatic skin of human patients

**Figure 1:**
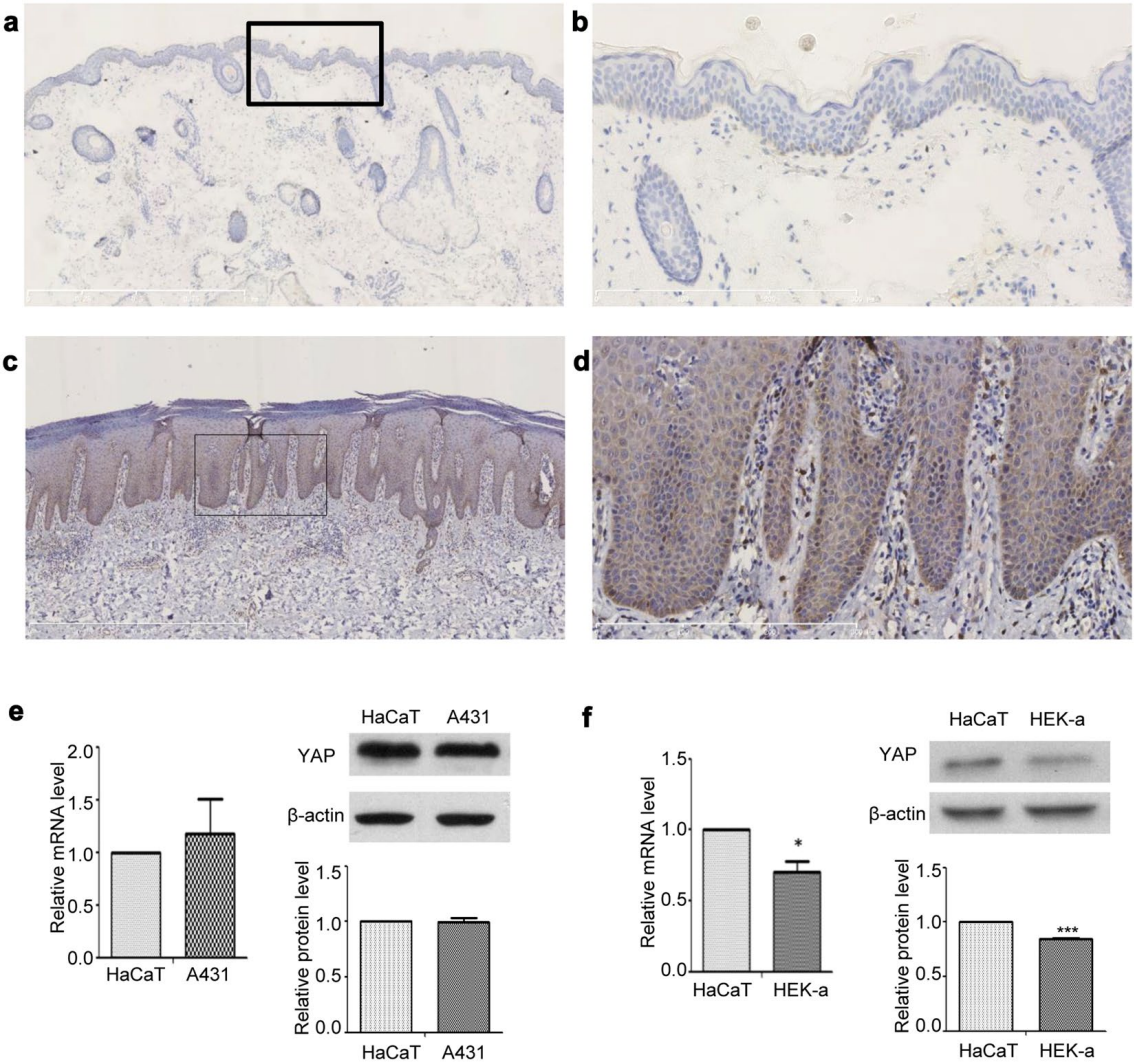
Immunohistochemical analysis of YAP expression in different tissues. (**a**) Normal skin (n = 30, bar length = 1 mm). (**b**) Magnification of the black box in image (a) (bar length = 300 μm). (**c**) Psoriasis (n = 33, bar length = 1 mm). (**d**) Magnification of the black box in image (**c**) (bar length = 300 μm). (**e**) qPCR analysis of YAP mRNA expression and Western blot analysis of YAP protein expression in the human immortalized epidermal keratinocyte cell line HaCaT and the cSCC cell line A431. (**f**) qPCR analysis of YAP mRNA expression and Western blot analysis of YAP protein expression in the HaCaT cell line and the human epidermal keratinocyte cell line HEK-a. *P < 0.05, ***P < 0.001.

We used immunohistochemistry to investigate YAP protein expression in the skin of 33 psoriasis patients and 30 normal controls. YAP was expressed in both nuclear and cytoplasmic locations. In normal control skin, YAP was weakly expressed in the stratum basale in 26.667% (8/30) of samples. In psoriatic skin, YAP was mainly expressed in the stratum basale and lower stratum spinosum in 54.545% (18/33) of the samples (Table 1 and Fig. 1a–d; examples of each staining intensity are shown in Supplementary Fig. S1). There was no staining in the stratum granulosum and stratum corneum. The difference in the positive staining rate between the psoriatic and normal control skin was statistically significant (χ2 = 5.039, *P* = 0.025). No staining was evident in when the isotype control, matched rabbit IgG isotype control antibody was substituted for the primary antibody (Supplementary Fig. S2a,b). Because the stratum basale is the germinal zone of the epidermis, these results suggest that increased expression of YAP may cause hyperproliferation of keratinocytes, directly contributing to the pathogenesis of psoriasis.

**Table 1:** Expression of YAP in normal skin and psoriatic tissues.

Group	n	Expression Grade, n				Positive rate (%)
		−	+	++	+++	
Normal skin	30	22	6	2	0	26.667
Psoriasis	33	15	6	9	3	54.545*

−Negative, score 0; +weakly positive, score 1–4; ++moderately positive, score 5–8; +++strongly positive, score 9–12.

*P < 0.05 compared with the control group.

### YAP expression is higher in HaCaT cells than in human primary keratinocytes

HaCaT cells are immortalized human epidermal keratinocytes, which are a commonly used cell model in psoriasis research^15–18^. HEK-a cells are primary human epidermal keratinocytes. A431 cells are cSCC cells, which are known to express high levels of YAP^12^. We used qPCR and Western blotting to compare YAP expression at the mRNA and protein levels among HaCaT, A431 and HEK-a cells. The level of YAP expression in HaCaT cells was similar to that in A431 cells, but was much higher than that in HEK-a cells (Fig. 1e,f). These results showed a direct correlation between increased keratinocyte proliferation and increased expression of YAP.

### YAP expression is upregulated in the psoriatic skin of mice with IMQ-induced psoriasis

**Figure 2:**
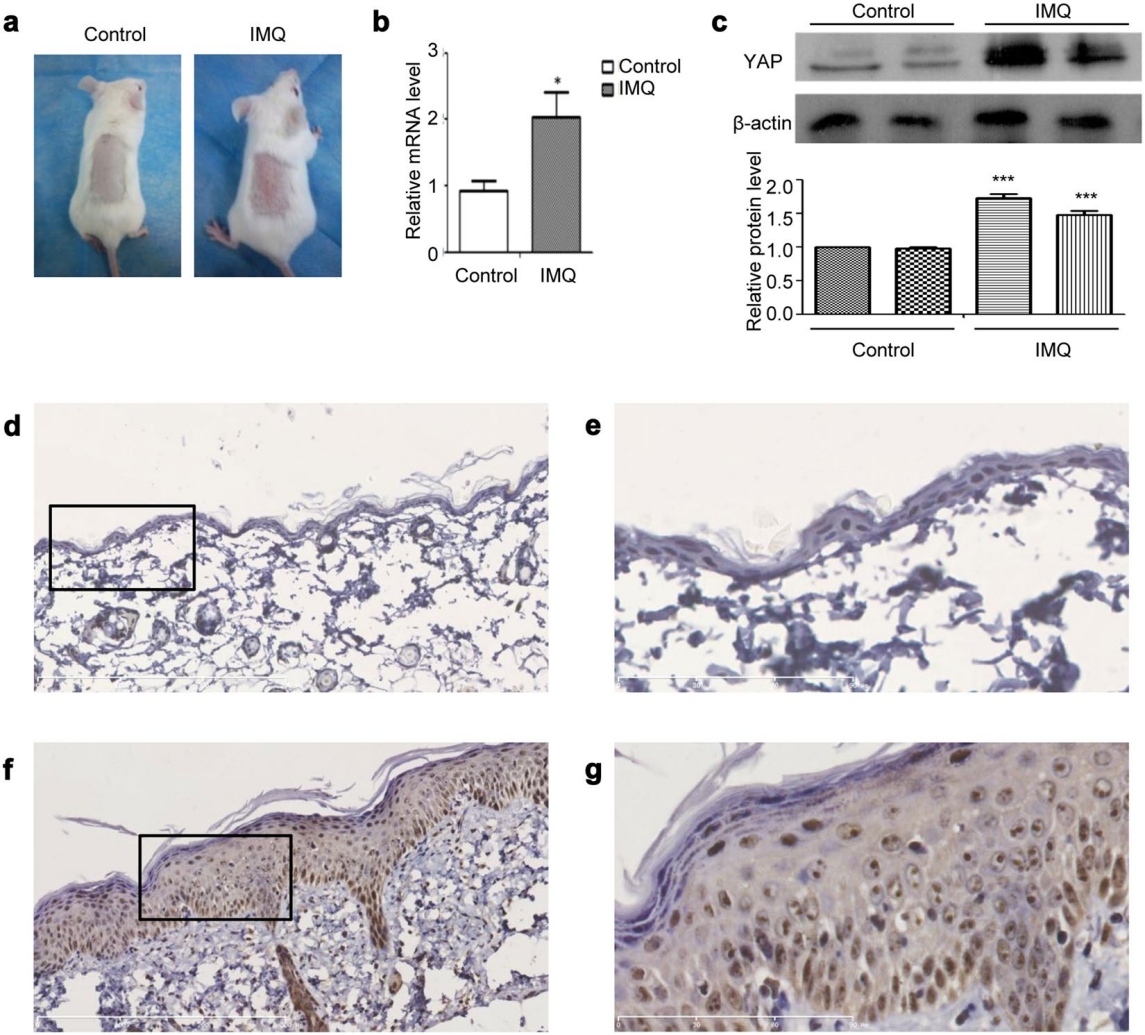
YAP expression in the IMQ mouse models. (**a**) Gross morphology of IMQ and control group tissues. (**b**) qPCR analysis of YAP mRNA expression in the IMQ and control groups. (**c**) Western blot analysis of YAP protein expression in the IMQ and control groups. (**d**) Immunohistochemical results for the control group (n = 6, bar length = 300 μm). (**e**) Magnification of the black box in image (**d**) (bar length = 90 μm). (**f**) Immunohistochemical results for the IMQ group. (n = 6, bar length = 300 μm). (**g**) Magnification of the black box in image (**f**) (bar length = 90 μm). *P < 0.05, ***P < 0.001.

Knowing that psoriasis patients’ skin and HaCaT cells express increased levels of YAP, we then determined whether mice with the psoriatic skin of IMQ-induced psoriasis (a widely used animal model of psoriasis) also exhibits increased YAP expression. After daily application of topical IMQ for 7 days, the IMQ-treated skin became thickened, with erythematous and scaled, in contrast to the skin treated with control cream (Fig. 2a). Immunohistochemistry showed negative or weak YAP staining in the stratum basale of the control group, with a positive rate of 16.667% (1/6), while the IMQ-induced psoriatic skin showed strong YAP staining, with a positive rate of 83.333% (5/6) (Table 2 and Fig. 2d–g). The difference between the two groups was statistically significant (χ2 = 5.333, *P* = 0.021). No staining was evident in when the isotype control, matched rabbit IgG isotype control antibody was substituted for the primary antibody (Supplementary Fig. S2c,d). By qPCR and Western blot analysis, we determined that levels of YAP mRNA and protein were also up-regulated in the IMQ group compared with the control group (Fig. 2b,c). These results demonstrated that YAP expression is also upregulated in the psoriatic skin of the IMQ-induced mouse model of psoriasis, further suggesting that increased levels of YAP in the skin cause increased proliferation of keratinocytes.

**Table 2:** Expression of YAP in IMQ mice and control mice.

Group	n	Expression Grade, n		Positive rate (%)
		−	+	
Control group	6	5	1	16.667
IMQ group	6	1	5	83.333*

*P < 0.05 compared with the control group.

### YAP knockdown inhibits the proliferation of HaCaT cells

**Figure 3:**
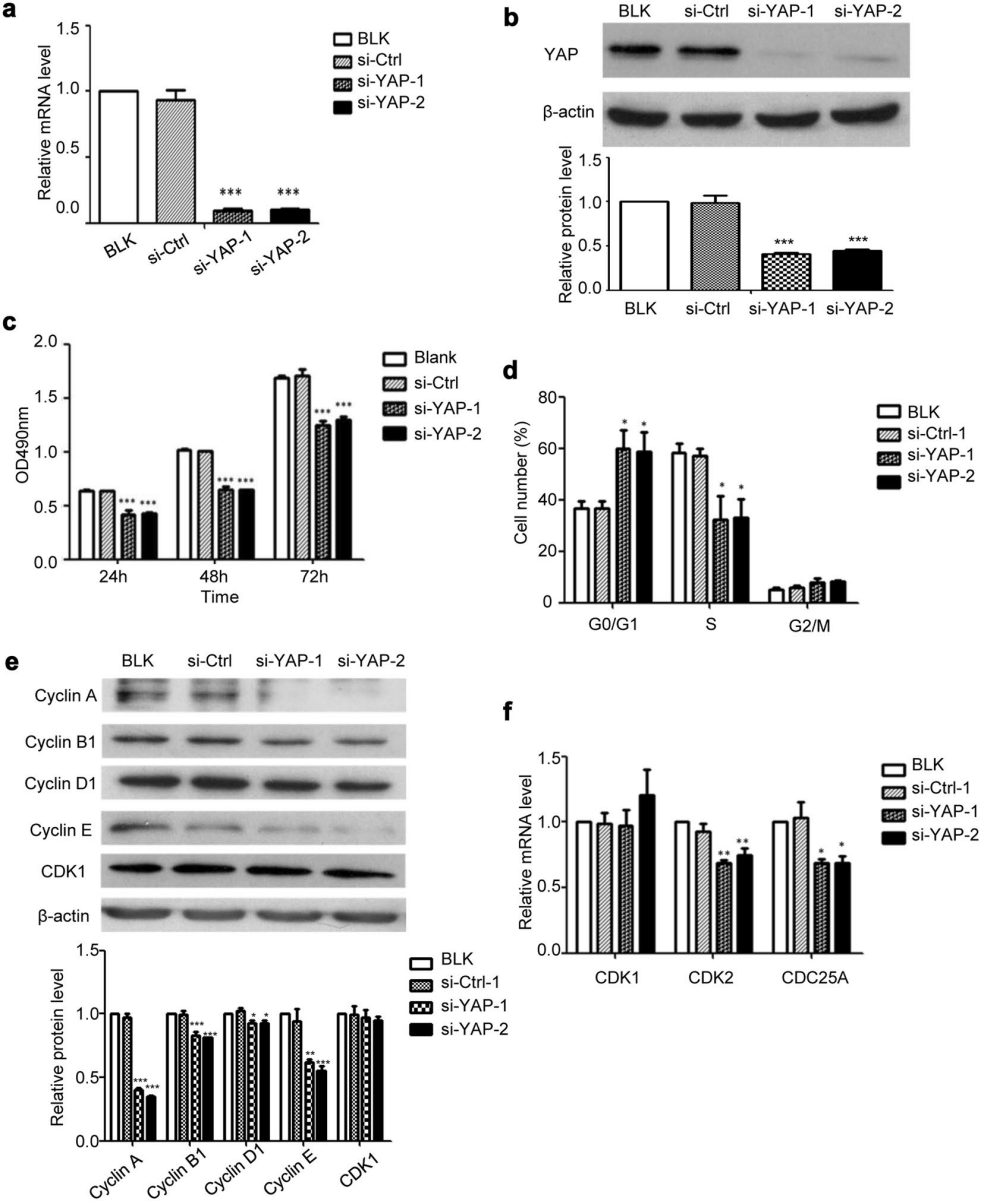
Effect of YAP downregulation on HaCaT cell proliferation and cell cycle. (**a**) YAP mRNA expression (24 h after transfection) was measured after YAP-siRNA transfection. (**b**) YAP protein expression (48 h after transfection) was measured after YAP-siRNA transfection. (**c**) The MTT assay was performed 24 h, 48 h, and 72 h after transfection. (**d**) The cell cycle profiles were analysed by flow cytometry 48 h after transfection. (**e**) The expression of cell cycle regulators was analysed by Western blotting after YAP knockdown. (**f**) The expression of cell cycle regulators were analysed by qPCR after YAP knockdown. All the quantitative data are presented as the mean ± standard error of the mean (n = 3). *P < 0.05, **P < 0.01, ***P < 0.001.

If the hyperproliferation of keratinocytes is due to the increased YAP level, then blockade of YAP expression should lead to reduced keratinocyte proliferation. To test this hypothesis, HaCaT cells were transfected with YAP-specific siRNAs (si-YAP-1 and si-YAP-2) or with control siRNA and analysed by qPCR 24 h after transfection to determine YAP mRNA levels, and analysed by Western blot 48 h after transfection to determine YAP protein levels. Both the mRNA and protein levels of YAP in HaCaT cells transfected with YAP-specific siRNAs were significantly lower than those in the non-transfected (BLK) and negative siRNA-transfected (si-Ctrl) groups (Fig. 3a,b).

More importantly, knockdown of YAP significantly inhibited the proliferation of HaCaT cells, as determined by the MTT assay (Fig. 3c).

### YAP knockdown causes G0/G1 phase arrest in HaCaT cells

To investigate the role of YAP in cell cycle regulation, a propidium iodide (PI) cell cycle analysis was performed in HaCaT cells 48 h after YAP knockdown. As shown in Fig. 3d, the proportion of cells in G0/G1 phase was significantly higher in the si-YAP groups than in the BLK and si-Ctrl groups, indicating that YAP knockdown led to growth arrest in the G0/G1 phase of the cell cycle. Further qPCR and Western blot analyses showed that the protein levels of cyclin A, cyclin B1, cyclin D1 and cyclin E, and the mRNA levels of CDK2 and CDC25A decreased by different degrees after YAP knockdown, but the mRNA and protein levels of CDK1 were not affected (Fig. 3e,f). Cyclin A-CDK2 complexes play an important role in S phase. Cyclin B plays a role in mitosis. Cyclin D and cyclin E play a role in early G1 phase and the G1/S phase transition^19^. The transition from G1 to S requires CDC25A^20^. Therefore, our results suggest that YAP can affect the HaCaT cell cycle progression by affecting the expression of downstream cell cycle regulators.

### YAP knockdown promotes apoptosis of HaCaT cells

**Figure 4:**
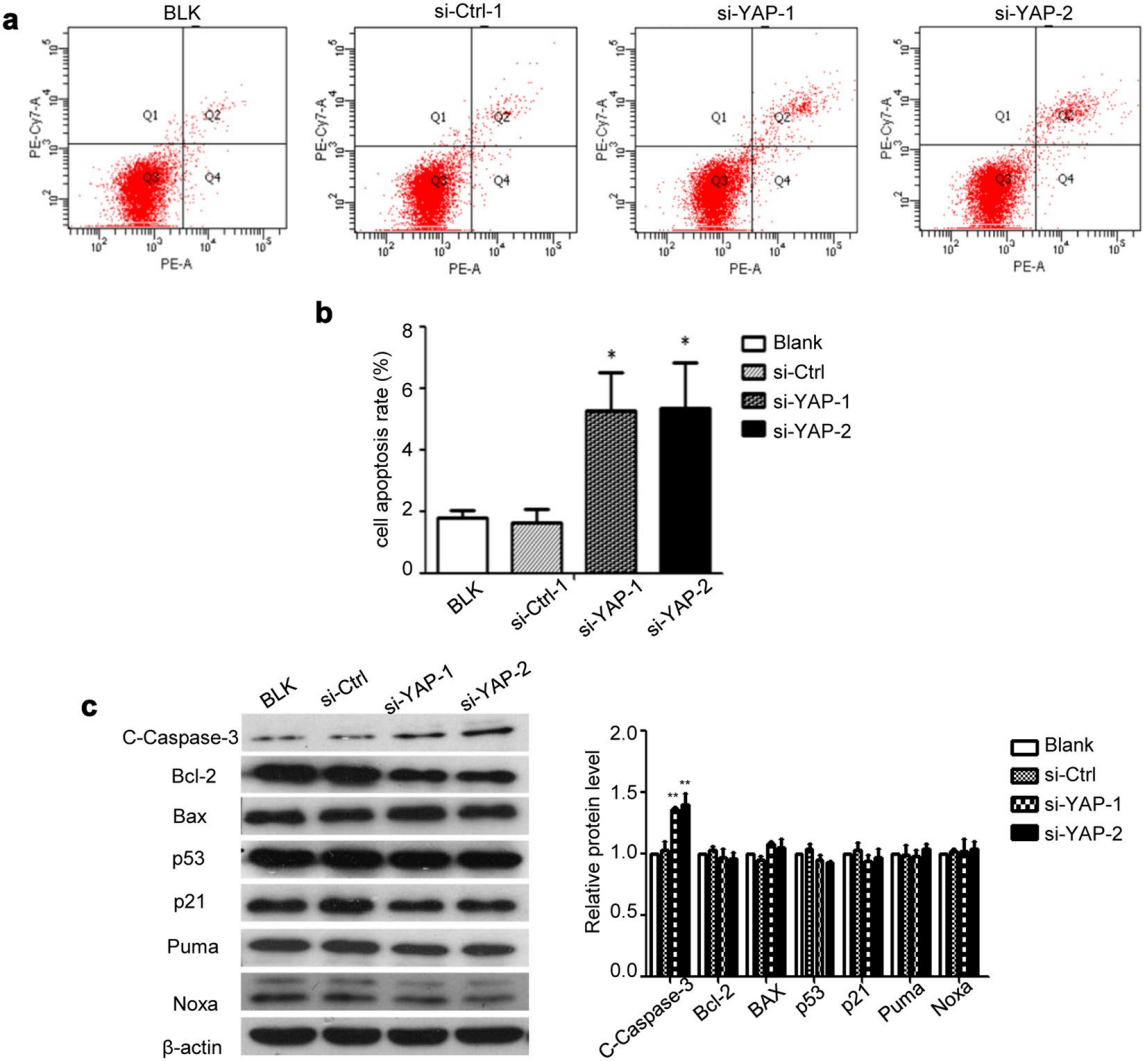
Effect of YAP downregulation on HaCaT cell apoptosis. HaCaT cells were transfected with YAP-siRNA and analysed 48 h later. (**a**) Cell apoptosis was analysed by annexin V/7-AAD staining and flow cytometry. (**b**) Quantification of the results in (**a**). (**c**) Transfected cells were lysed for Western blot analysis of indicated proteins. All the quantitative data are presented as the mean ± standard error of the mean (n = 3). *P < 0.05, **P < 0.01.

To test whether YAP plays any role in regulating cell apoptosis, we quantified apoptosis in HaCaT cells with vs. without YAP knockdown using 7-AAD/PE Annexin V staining. Cells in the BLK and si-Ctrl groups showed a low basal apoptosis rate (1.800 ± 0.208% and 1.633 ± 0.418%, respectively). YAP knockdown, however, increased the apoptosis rate to 5.267 ± 1.241% and 5.333 ± 1.477% in the si-YAP-1 and si-YAP-2 groups, respectively (Fig. 4a,b). Further Western blot analysis showed that the protein level of cleaved caspase-3 (C-caspase-3), a marker of apoptosis, was elevated, while the levels of Bcl-2, BAX, and p53 and its down stream targets p21, Puma and Noxa were not affected (Fig. 4c).

### AREG and other signalling pathways are involved in the YAP-mediated effects on cell proliferation in HaCaT cells

**Figure 5:**
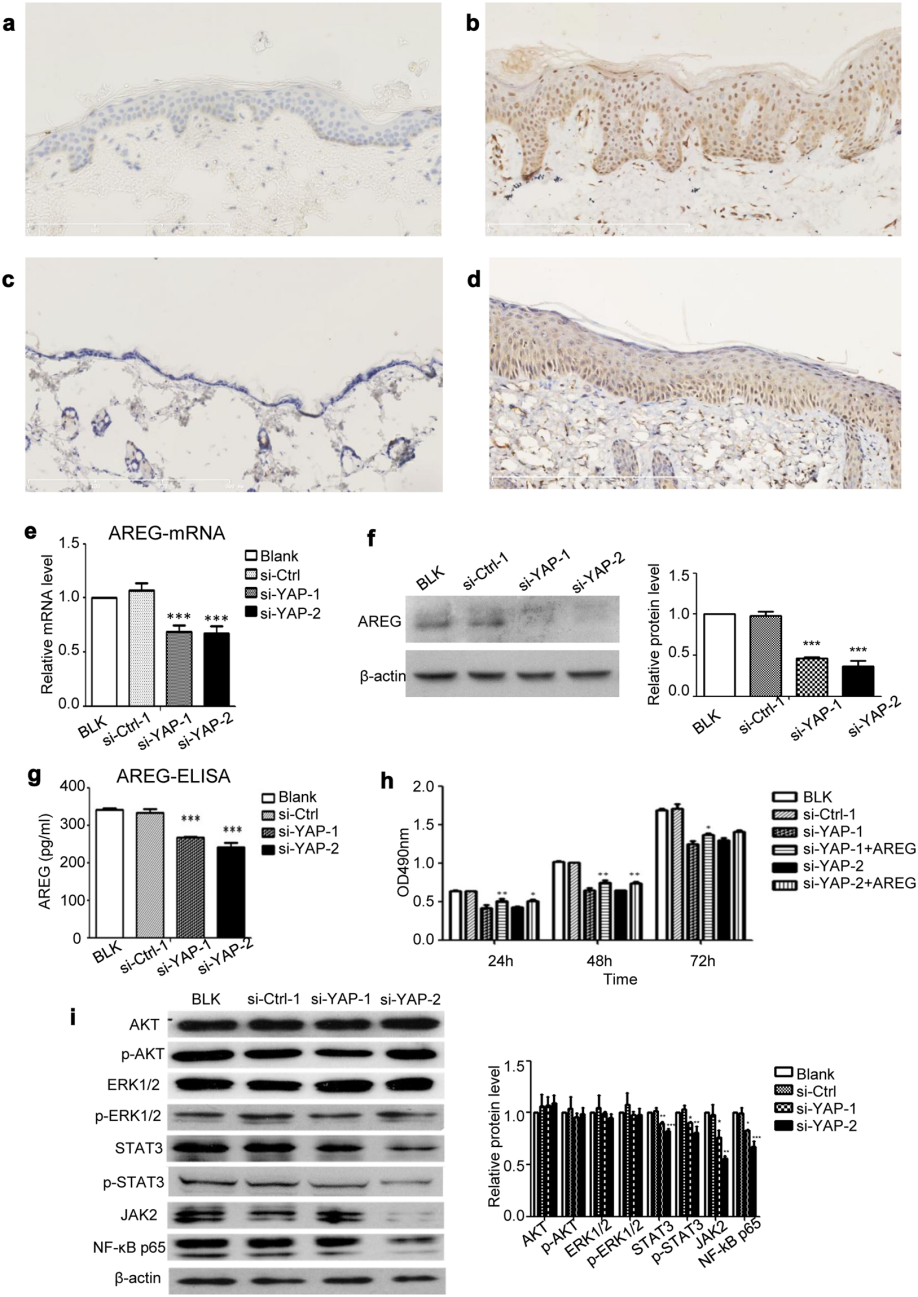
AREG and other signalling pathways are involved in the YAP-mediated regulation of cell proliferation in HaCaT cells. (**a**) Immunohistochemical results for normal skin (n = 30, bar length = 300 μm) and (**b**) psoriatic skin (n = 33, bar length = 300 μm). (**c**) Immunohistochemical results for control mice (n = 6, bar length = 300 μm) and (**d**) mice with IMQ-induced psoriasis (n = 6, bar length = 300 μm). (**e**) qPCR analysis of AREG mRNA expression 24 h after YAP knockdown. (**f**) Western blot analysis of cellular AREG protein 48 h after YAP knockdown. (**g**) ELISA assays measuring the concentration of secreted AREG 48 h after YAP knockdown in conditioned medium. (**h**) MTT assays of control or YAP-depleted HaCaT cells in the presence or absence of AREG. (**i**) The expression of AKT, phospho-AKT, ERK1/2, phospho-ERK1/2, STAT3, phospho-STAT3, JAK2 and NF-κB p65 were analysed by Western blotting 48 h after transfection with YAP-siRNA. All the quantitative data are presented as the mean ± standard error of the mean (n = 3). *P < 0.05, **P < 0.01, ***P < 0.001.

AREG, a ligand for EGFR, plays an important role in cell growth^21^. AREG promoted the proliferation of HaCaT cells in a dose-dependent manner in culture medium with or without serum (Supplementary Fig. S3). Our results showed that in the human samples, AREG could be found in 51.515% (17/33) of the psoriatic skin samples, while and in 20.000% (6/30) of the normal skin samples (Table 3 and Fig. 5a,b). The difference in the positive staining rate of AREG between the psoriatic and normal control skin was statistically significant (*χ2* = 6.733, *P* = 0.009). Furthermore, the relationship between YAP and AREG histoscores showed a significant positive correlation (*r* = 0.958, *P* < 0.001). In the mouse samples, AREG could be found in 66.667% (4/6) of the samples from the IMQ-induced psoriasis group and in 16.667% (1/6) of the control samples (Table 4 and Fig. 5c,d). Although the difference in the positive staining rate of AREG between the IMQ-induced psoriatic skin and control skin was obvious, and the relationship between YAP and AREG expression showed a strong positive correlation, neither of these differences reached statistical significance (*χ2* = 3.086, *P* = 0.079; *r* = 0.894, *P* = 0.106), probably due to the limited animal sample size. These results indicated that AREG may be also be involved in the pathogenesis of psoriasis, and that AREG was positively related to YAP. In the *in vitro* studies, the levels of AREG mRNA (Fig. 5e), intracellular AREG protein (Fig. 5f) and secreted AREG protein (Fig. 5g) in HaCaT cells were reduced after YAP knockdown. In addition, AREG depletion by specific siRNAs resulted in the inhibition of HaCaT cell proliferation. However, the addition of 100 nmol/L AREG to the si-YAP-transfected HaCaT cells partially restored the cell growth (Fig. 5h), suggesting that YAP regulates cell proliferation through the regulation of AREG expression. Using Western blot analysis, we examined the impact of YAP knockdown on other key signalling pathway molecules. YAP knockdown inhibited STAT3, JAK2 and NF-κB p65 to different degrees but had no effect on p-AKT and p-ERK in HaCaT cells (Fig. 5i).

**Table 3:** Expression of AREG in normal skin and psoriasis tissues.

Group	n	Expression Grade, n				Positive rate (%)
		−	+	++	+++	
Normal skin	30	24	5	1	0	20.000
Psoriasis	33	16	9	6	2	51.515**

−Negative, score 0; +weakly positive, score 1–4; ++moderately positive, score 5–8; +++strongly positive, score 9–12.

**P < 0.01 compared with the control group.

**Table 4:** Expression of AREG in IMQ mice and control mice.

Group	n	Expression Grade, n		Positive rate (%)
		−	+	
Control group	6	5	1	16.667
IMQ group	6	2	4	66.667

## Discussion

Recently, increasing evidence has implicated the oncogene YAP in the pathogenesis of many human cancers^22–24^, since it promotes cell proliferation, regulates the cell cycle, inhibits apoptosis and promotes the migration and invasion of tumour cells. Keratinocyte hyperproliferation and shortened cell cycles are important pathophysiological features of psoriasis, and psoriatic keratinocytes are resistant to apoptosis. These features of psoriatic keratinocytes are similar to those of tumour cells. Therefore, tumour-related regulatory factors and signalling pathways have attracted widespread attention in the study of psoriasis in recent years^25–28^. Complicated immune networks may be involved in the mechanisms of keratinocyte hyperproliferation, but the details remain unclear. Studies have shown that, immune cells such as CD4+ T cells, CD8+ T cells and Th17 cells, cytokines such as IL-1, IL-6, TNF-α, IL-17, IL-22 and IL-23, and antimicrobial peptides such as LL-37 and psoriasin could lead to the development of inflammation^29^ and activate different signalling pathways such as the STAT3, NF-κB, AKT and ERK^30–34^ pathways, which finally results in the abnormal proliferation of keratinocytes. Because abnormal hyperproliferation is the final manifestation of various inflammatory pathways, it is important to study the factors that may be at fault. We previously showed that YAP was up-regulated in cSCC tissues and cell lines compared to normal controls, and its expression had an increasing trend from normal skin → precancerous lesions of actinic keratosis (AK) → carcinoma *in situ* (Bowen’s Disease) → well-differentiated cSCC → moderately and poorly differentiated cSCC^12^. Downregulation of YAP in cSCC cells inhibited the expression of the cell cycle regulators CDK2, CDC25A, cyclin A, cyclin B1, cyclin D1 and cyclin E, causing G0/G1 cell cycle arrest and increased apoptosis, possibly through the AREG/RAS/AKT/ERK pathways^12^. Because psoriasis shares the common feature of abnormal keratinocyte proliferation and similar signalling pathways with cSCC, it is readily inferred that YAP may play a similar role in the progression of psoriasis as it does in cSCC.

The present results show significantly increased expression of YAP in clinical psoriatic specimens and in specimens from the IMQ-induced mouse model of psoriasis. D’Addario *et al*.^35^ found that overexpression of YAP in normal human primary keratinocytes interfered with their normal differentiation process and led to immortalized proliferation, upregulation of the epithelial proliferation markers p63 and PCNA, and downregulation of the differentiation markers 14-3-3σ and LEKTI. In HaCaT cells, an immortalized epidermal keratinocyte cell line widely used as a cell model of psoriasis^15–18^, we found a high level of YAP that almost reached the level of YAP in the cSCC cell line A431 and was significantly higher than that in primary keratinocytes. Further functional analyses of HaCaT cells showed that, similar to its effects in cSCC, YAP promotes the proliferation, regulates the cell cycle and inhibits the apoptosis of HaCaT cells through modulating cell cycle regulators and apoptosis-related proteins. These results confirm that psoriasis and cSCC share some common pathological mechanisms. However, unlike in cSCC, in which YAP functions through the AKT and ERK pathways, YAP may function through the STAT3, JAK2 and NF-κB pathways in psoriasis. Further studies are needed to identify the specific regulatory sites for YAP activity in psoriasis.

AREG is overexpressed in a variety of human cancers, such as colon, breast, lung, liver, prostate, gastric, cancer and pancreatic^36^. In some abnormal epidermal proliferative skin diseases such as psoriasis, AK, warts, keratoacanthoma and cSCC, the expression of AREG is also increased. Overexpression of AREG in HaCaT cells enhances cell proliferation^13,14^. These findings indicate that the proliferative ability of cells is correlated with the expression level of AREG. It is generally believed that AREG is a downstream transcriptional target of YAP^37^. Zhang *et al*.^38^ found that YAP played a role in the phosphorylation of the AREG downstream molecules AKT and ERK, and that knockdown of AREG blocked this effect, suggesting that YAP promotes the expression of AREG. AREG then binds to and activates EGFR, leading to subsequent activation of its downstream signalling pathways. Our study showed that AREG expression was positively correlated with that of YAP in both human and mouse samples and could promote the proliferation of HaCaT cells in a dose-dependent manner. The secretion of AREG was reduced in YAP-depleted HaCaT cells. Adding 100 nmol/L AREG back to the YAP knockdown cells partially restored the growth rate. Therefore, AREG is involved in the YAP-mediated regulation of cell proliferation, cell cycle and apoptosis in psoriatic keratinocytes.

In conclusion, the findings from our study suggest that YAP plays an important role through AREG in the abnormal proliferation and differentiation of keratinocytes in psoriasis. Targeting the YAP-mediated pathways may be have therapeutic efficacy in the treatment of psoriasis.

## Materials and Methods

### Patient samples

Patient samples were obtained between Jul 2015 and Sep 2016 from the tissue bank of the Department of Dermatology at the Second Affiliated Hospital of Xi’an Jiaotong University. A total of 63 samples were collected, including 33 psoriatic tissues (from 17 males and 16 females, age range = 10–77 years) and 30 normal skin tissues (from 19 males and 11 females, age range = 11–63 years); the samples were obtained from cosmetic surgery. Written informed consent for tissue collection and use was obtained from all adult patients or the parents/legal guardians for patients under 18 years before the study, and an ethics approval was obtained from the Institutional Ethics Committee of Xi’an Jiaotong University.

### Immunohistochemistry

Immunohistochemical staining was performed by a standard immunoperoxidase staining procedure^39^. Two pathologists independently observed the results under a microscope. The results were quantified based on the following scoring system: the first score was obtained based on the percentage of positive cells (≤5% = 0, 6–25% = 1, 26–50% = 2, 51–75% = 3, and >75% = 4). The second score was obtained based on the staining intensity (colourless = 0, light yellow = 1, yellowish-brown = 2, and chocolate brown = 3). The overall score of each microscopic field was calculated as the product of the two scores, and the final score was the average of the score in five fields.

### Cell culture

The human immortalized epidermal keratinocyte cell line HaCaT and the cSCC cell line A431 were obtained from ATCC (USA) through an authorized local ATCC dealer (Xiangf Biotechnology, Shanghai, China) and were maintained in Dulbecco’s modified Eagle’s medium (Gibco/Thermo Fisher Scientific, CA, USA). The human epidermal keratinocyte cell line HEK-a was from ScienCell Research Laboratories (CA, USA) and was maintained in keratinocyte medium (ScienCell Research Laboratories). The culture media were supplemented with 10% foetal bovine serum (FBS), penicillin (100 units/ml), and streptomycin (100 mg/ml). Cells were routinely cultured in a humidified incubator at 37 °C and 5% CO_2_.

### Transient transfection of siRNA

The siRNA oligonucleotide sequence (shown in Supplementary Table S1) was synthesized by Shanghai GenePharma (Shanghai, China). Twenty-four hours before transient transfection, the cells were seeded at a density of 2 × 10^5^ cells/well in 6-well plates. When the confluence reached 70–80%, the cells were transfected with siRNA according to the recommended procedures for LipofectamineTM2000 Transfection Reagent (Invitrogen, Carlsbad, CA).

### Quantitative Real-Time PCR

Twenty-four hours after transfection, total RNA was extracted from the cells using TRIzol reagent (Invitrogen, Carlsbad, CA) and was then reverse transcribed to complementary DNA (cDNA). The primers (sequences shown in Supplementary Table S2) were synthesized by Sangon Biotech (Shanghai, China). SYBR premix EX Taq I (Takara, Japan) was used as a DNA-specific fluorescent dye, and the results were analysed by a 7500 real-time PCR system (Applied Biosystems, Foster City, CA) through the comparative Ct (ΔCt) method. The experiments were repeated three times, and all three technical replicates were included for each data point.

### Western blot analysis

The following antibodies were purchased from Cell Signaling Technology (Danvers, MA, USA): YAP (#4912), AKT (pan) (#4691), ERK 1/2 (#9102), phospho-AKT (#2965), phospho-ERK 1/2 (#4376), cleaved caspase-3 (#9664), Bcl-2 (#2870), BAX (#2872) and phospho-STAT3 (#9145). Antibodies against cyclin A (sc-596), cyclin B1 (sc-595), cyclin D1 (sc-246), cyclin E (sc-247), p53 (sc-126) and β-actin (sc-47778) were purchased from Santa Cruz Biotechnology (Dallas, TX, USA). The CDK1 (cdc2) antibody(19532-1-AP) was purchased from Proteintech (Chicago, IL, USA). The STAT3 antibody(wl0836) was from Wanleibio (Shengyang, China). The Puma (ab33906), Noxa (ab140129), JAK2 (ab108596) and NF-κB p65 (ab19870) antibodies were from Abcam (Cambridge, UK). AREG antibody (AF262) was from R&D Systems (Minneapolis, MN, USA). Protein expression levels were analysed by standard Western blotting protocols.

### MTT assay for cell proliferation analysis

Cell proliferation was assessed using an MTT assay. The cells were seeded at a density of 5 × 10^3^ cells/well in 96-well plates and transfected with siRNA the next day in the presence or absence of recombinant AREG (R&D Systems, Minneapolis, MN, USA). After the cells were cultured for another 24 h, 48 h, or 72 h, 0.5 mg/ml MTT (Sigma-Aldrich, Germany) was added, and the cells were cultured for an additional 4 h. Then, the supernatant was removed, 150 µl of dimethyl sulfoxide (DMSO, Sigma-Aldrich, Munich, Germany) was added to each well, and the plates were placed on a shaker for 10 min. The optical density (OD) was measured at 490 nm using a microplate reader (Bio-Rad). The mean readings from three independent experiments were plotted for each time point. Five technical replicates were performed for each data point.

### PI staining for cell cycle analysis

Forty-eight hours after transfection, the cells were harvested, fixed in 70% ethanol, and stored overnight at −20 °C. For the analysis, PI staining solution (50 μg/mL PI and 100 μg/mL RNase A) was added to the cells, which were then incubated for 30 min in the dark at 37 °C. The cells were analysed using flow cytometry (FACSCalibur, BD Biosciences, USA). The Modifit version 3.3 software (Verity Software House, USA) was used to analyse the results. Three independent experiments were performed.

### Annexin V/7-AAD staining for cell apoptosis analysis

The siRNA-transfected cells were harvested 48 h after transfection. For the analysis, the cells were stained with the PE Annexin V Apoptosis Detection Kit I (BD Biosciences, USA) following the manufacturer’s instruction. The results were analysed using flow cytometry (FACSCalibur, BD Biosciences, USA). Three independent experiments were performed.

### AREG ELISA

AREG protein levels were calculated by a Human Amphiregulin Quantikine ELISA Kit from R&D Systems following the manufacturer’s instructions. Three independent experiments were performed, and three technical replicates were included for each data point.

### IMQ mouse models

Female BALB/c mice (6–8 weeks old) were purchased from Beijing Vital River Company (Beijing, China). The mice were shaved approximately 2 * 2 cm on the back and were then randomly divided into a normal control group and an IMQ model group (6 mice in each group). The IMQ group received a daily topical application of 62.5 mg of 5% IMQ cream (Sichuan Med-Shine Pharmaceutical Co., Ltd; China), and the control group received the same dosage of Vaseline. After 7 days, the mice were sacrificed, and the skin lesions were removed. A portion of the tissues was fixed with 4% paraformaldehyde and embedded in paraffin for further immunohistological studies, and other portions were stored in liquid nitrogen for quantitative real-time PCR and Western blot analysis. All animal protocols were approved by the Institutional Animal Care and Use Committee of Xi’an Jiaotong University.

### Statistical analysis

All data are presented as the mean ± standard error. Statistical analysis was carried out using the SPSS statistical package (SPSS, Chicago, IL, USA). The Pearson chi-square test was used for the immunohistochemistry analysis. The Spearman correlation coefficient was used to determine the correlation between YAP and AREG. Student’s t-test was used for comparisons between two groups, and one-way analysis of variance was used for experiments with more than two groups. A *P* value of less than 0.05 was considered statistically significant.

All methods were performed in accordance with the relevant guidelines and regulations.

## Electronic supplementary material

Dataset 1
