# Supplementary material for: Yes-associated protein promotes the abnormal proliferation of psoriatic keratinocytes via an amphiregulin dependent pathway
Source: Sci Rep. 2018 Oct 15;8:14513. doi: 10.1038/s41598-018-32522-y (PMC6189173; doi:10.1038/s41598-018-32522-y)
Supplement: Supplementary file 1 — Dataset 1 [file 41598_2018_32522_MOESM1_ESM.docx]

**Yes-associated protein promotes the abnormal proliferation of psoriatic keratinocytes via an amphiregulin dependent pathway**

Jinjing Jia^1,2^, Changji Li^2^, Jiao Yang^2^, Xin Wang^2^, Ruilian Li^2^, Suju Luo^3^, Zhengxiao Li^2^, Jiankang Liu^4,5^, Zhi Liu^6^, Yan Zheng^2*^

^1^Department of Dermatology, the Second Affiliated Hospital of Guangzhou University of Chinese Medicine, Guangzhou 510120, China

^2^Department of Dermatology, Second Affiliated Hospital of Xi’an Jiaotong University, School of Medicine, Xi’an, China

^3^Department of Dermatology, Tianjin Medical University General Hospital, Tianjin, China

^4^Key Laboratory of Biomedical Information Engineering of the Ministry of Education, School of Life Science and Technology, Xi’an Jiaotong University, Xi’an, China

^5^Frontier Institute of Science and Technology, Xi’an Jiaotong University, Xi’an, China

^6^Department of Dermatology, University of North Carolina at Chapel Hill, Chapel Hill, NC 26599, USA

**Corresponding author:**

Dr. Yan Zheng. E-mail: [doctor_zhengyan@163.com](mailto:doctor_zhengyan@163.com)

**Table S1.** The sequences of siRNAs used in this study

| Name | Sequences |
| --- | --- |
| YAP-1 | Forward: 5’- GACAUCUUCUGGUCAGAGATT -3’,  Reverse: 5’- UCUCUGACCAGAAGAUGUCTT -3’ |
| YAP-2 | Forward: 5’- CUGCCACCAAGCUAGAUAATT -3’  Reverse: 5’- UUAUCUAGCUUGGUGGCAGTT -3’ |
| Negative control-1 | Forward: 5’- UUCUUCGAACGUGUCACGUTT -3’  Reverse: 5’- ACGUGACACGUUCGGAGAATT -3’ |
| Negative control-2 | Forward: 5’- GCGACGAUCUGCCUAAGAUTT -3’  Reverse: 5’- AUCUUAGGCAGAUCGUCGCTT -3’ |

**Table S2.** The qRT-PCR primers used in this study

| Gene name | Sequences |
| --- | --- |
| human-YAP | Forward: 5’-CCTGCGTAGCCAGTTACCAA-3’  Reverse: 5’-CCATCTCATCCACACTGTTC-3’ |
| mouse-YAP | Forward: 5’-TCCGAAATCTTGGACGTGGA-3’ |
|  | Reverse: 5’-GGCAGAGAAAAGCGGAACAA-3’ |
| CDK1 | Forward: 5’- GTCCGCAACAGGGAAGAACAG-3’  Reverse: 5’-CGAAAGCCAAGATAAGCAACTCC-3’ |
| CDK2 | Forward: 5’-TGAAGATGGACGGAGCTTGTTAT-3’  Reverse: 5’-CTTGGTCACATCCTGGAAGAAAG-3’ |
| CDC25A | Forward: 5’-ACAGCTCCTCTCGTCATGAGAAC-3’  Reverse: 5’-GGTCTCTTCAACACTGACCGAGT-3’ |
| AREG | Forward: 5’-GGGAGTGAGATTTCCCCTGT-3’  Reverse: 5’-AGCCAGGTATTTGTGGTTCG-3’ |
| GAPDH | Forward: 5’-ACCACAGTCCATGCCATCAC-3’ |
|  | Reverse: 5’-TCCACCACCCTGTTGCTGTA-3’ |


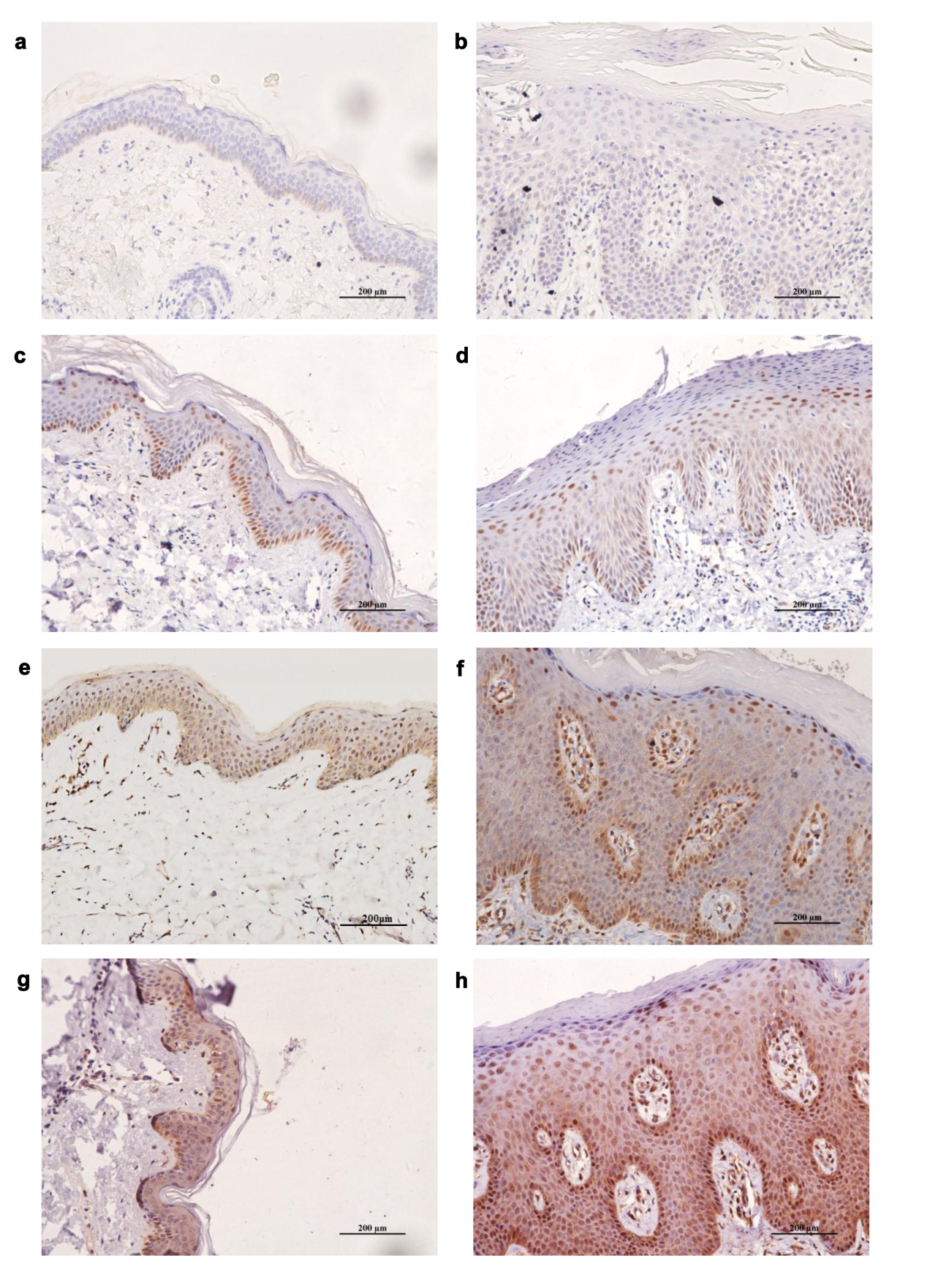


**Figure S1.** Immunohistochemistry of each staining intensity of YAP in different tissues. **(a) (c) (e) (g)** Examples of each staining intensity (from – to +++) in normal skin tissues. **(b) (d) (f) (h)** Examples of each staining intensity (from – to +++) in psoriatic tissues. Bar length = 200 μm.


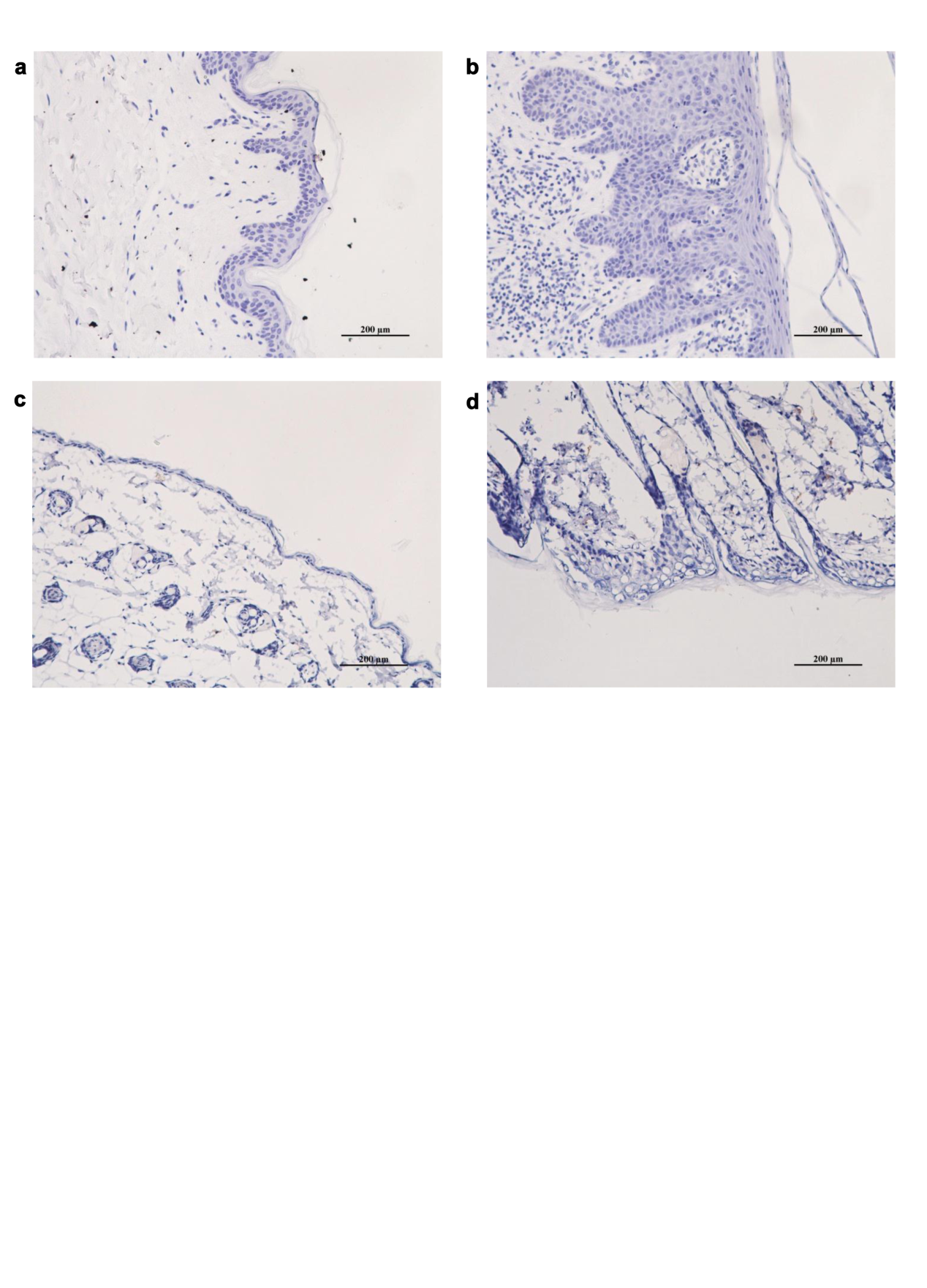


**Figure S2.** Immunohistochemistry of isotype control in clinical tissues and mouse models. **(a)** Normal skin tissues. **(b)** Psoriatic tissues. **(c)** Mice of control group. **(d)** Mice of IMQ-induced psoriasis group.


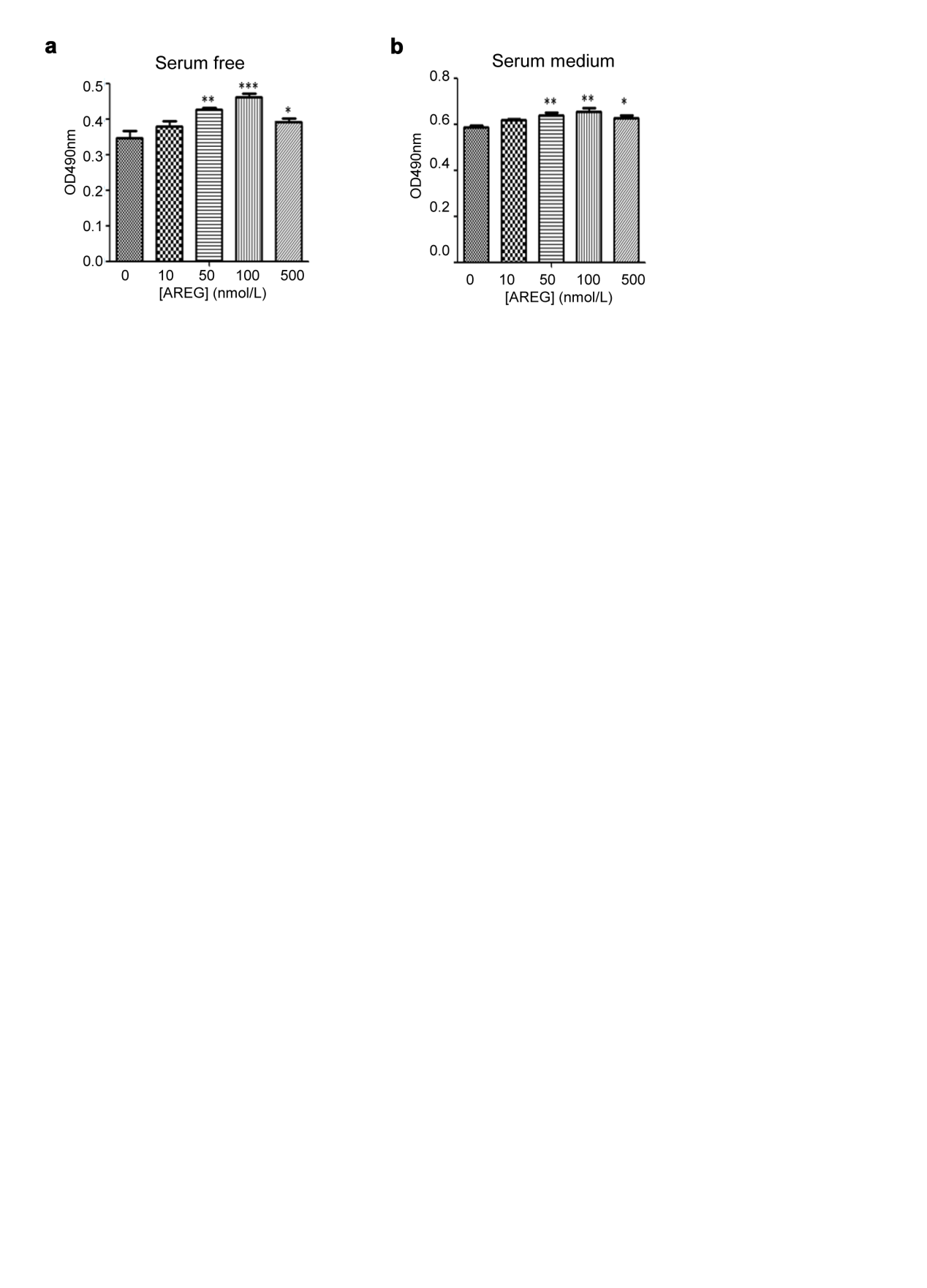


**Figure S3.** AREG promoted the proliferation of HaCaT cells. **(a)** The cells were serum-starved for 12 h and then treated with increasing concentrations of AREG (without serum) for 24 h. Cell growth was measured by MTT assays. **(b)** Same as (a) except that normal culture medium (with 10% FBS) was used.
